# Profiloplasty and facial contouring with injectables: A holistic, practical, and user‐friendly approach

**DOI:** 10.1111/jocd.16375

**Published:** 2024-05-20

**Authors:** Lisandro Farollch‐Prats, André Braz, Fernando Urdiales‐Gálvez, Carola de la Guardia, Ahmad Nazari

**Affiliations:** ^1^ Nu Clinic Barcelona Spain; ^2^ Dermatología Láser Cosmiatria Clinic Rio de Janeiro Brazil; ^3^ Instituto Médico Miramar Málaga Spain; ^4^ Global Aesthetics Medical Affairs, Allergan Aesthetics, an AbbVie Company Madrid Spain; ^5^ I‐Face Academy Istanbul Turkey

**Keywords:** botulinumtoxinA, dermal filler, full‐face approach, hyaluronic acid, profiloplasty

## Abstract

**Background:**

Expansion of the aesthetic treatment armamentarium with novel injectable products has greatly improved our ability to reshape and enhance the facial profile. However, full‐face “profiloplasty” with injectables remain a nascent concept and is insufficiently considered in daily practice. While techniques have been widely published for treating the individual facial areas that constitute an attractive profile—including the forehead, nose, lips, jawline, and chin—the literature remains fragmented on more universal methods for profile improvement.

**Aims:**

To collate disparate knowledge on profile optimization with injectables, and define a holistic, practical, and user‐friendly approach to profiloplasty and facial contouring.

**Methods:**

Consideration is given to key anatomical aspects and the changes that occur with age; patient assessment parameters for planning profiloplasty; and the technical specifications, product selection, and essential safety considerations for minimizing the risk of complications and optimizing the effectiveness of treatment.

**Results:**

Relevant case studies are presented.

**Conclusions:**

Holistic approaches of this type are essential if we are to maximize the potential of full‐face treatment with injectables—both now and in the future.

## 1 INTRODUCTION

The full‐face approach is increasingly becoming the standard of care when using injectables in aesthetic medicine. However, patients are usually evaluated primarily in frontal view, and the profile may be somewhat overlooked—or at least not assessed in detail. Practitioners risk missing important information if they inadequately consider this view. Moreover, patients frequently present looking for correction of problems that are relevant to the profile, such as nasal hump, inadequate lip projection, or chin retrusion.

The concept of full‐face “profiloplasty” with injectables is still nascent, and few papers have been published on this topic.^1^, ^2^, ^3^ Thus, while treatments of the various facial areas that constitute an attractive profile—such as the forehead, nose, lips, jawline, and chin—are widely covered, the literature remains fragmented on more holistic profile improvement.

Bertossi and colleagues have proposed a very useful algorithm for profiloplasty with injectables, and this provides details of treatment areas, injection techniques, and product recommendations.^1^ However, their work was primarily focused on individual treatment areas, and did not go into detail on facial assessment or key anatomical considerations.

Hence, there remains a need to collate the wealth of disparate knowledge on profile optimization with injectables and define more holistic approaches. These should consider underlying facial anatomy and the changes that occur with age; the most important assessment parameters when planning profiloplasty; and the technical specifications, product selection, and key safety aspects for minimizing the risk of complications and optimizing effectiveness. In this paper, we will attempt to define a practical, user‐friendly approach to profiloplasty and facial contouring that brings together all of these aspects. Consideration will be given both to individual treatment areas (moving down the face from forehead to chin) and to the profile as a whole.

## 2 FOREHEAD

### 2.1 Anatomy

The anatomy of the forehead has long been less well understood than many other facial areas. However, recent imaging studies have begun to shed light on the aging process of the forehead. Broadly speaking, when viewed laterally, it loses its convex shape over time and takes on a more concave profile.^4^ Computed tomography analyses have demonstrated that there are also more complex alterations in forehead shape that result from age‐related bone remodeling—including calvarial volume loss.^5^, ^6^

From a clinical perspective, the likely effects of these changes include greater laxity and altered draping of the soft tissues.^5^ Furthermore, lateral expansion can increase the apparent prominence of the lateral orbital rims, temporal crests, and zygomatic arches.^5^

In addition, multimodal assessments have shown that the skin in this area becomes firmer and less elastic with age—most likely due to loss of elastic types of collagen and alterations in overall protein composition—with associated increases in vertical skin mobility.^7^

Safe treatment practice requires a comprehensive understanding of the underlying anatomical layers. A recent study using ultrasound imaging and cadaveric dissection demonstrated the presence of eight such layers in the forehead (Figure 1).^8^ This work also identified a dense, robust mesh of fat and connective tissue located between the orbicularis retaining ligament, inferior frontal septum, subfrontalis fascia, and periosteum, which the authors termed “preperiosteal fat within the prefrontal space” (see layer 7 in Figure 1). With increasing age, this area may be prone to volume loss and descent of soft tissue, leading to loss of support in the lateral eyebrow, potentially causing lateral brow ptosis.^8^

**FIGURE 1:**
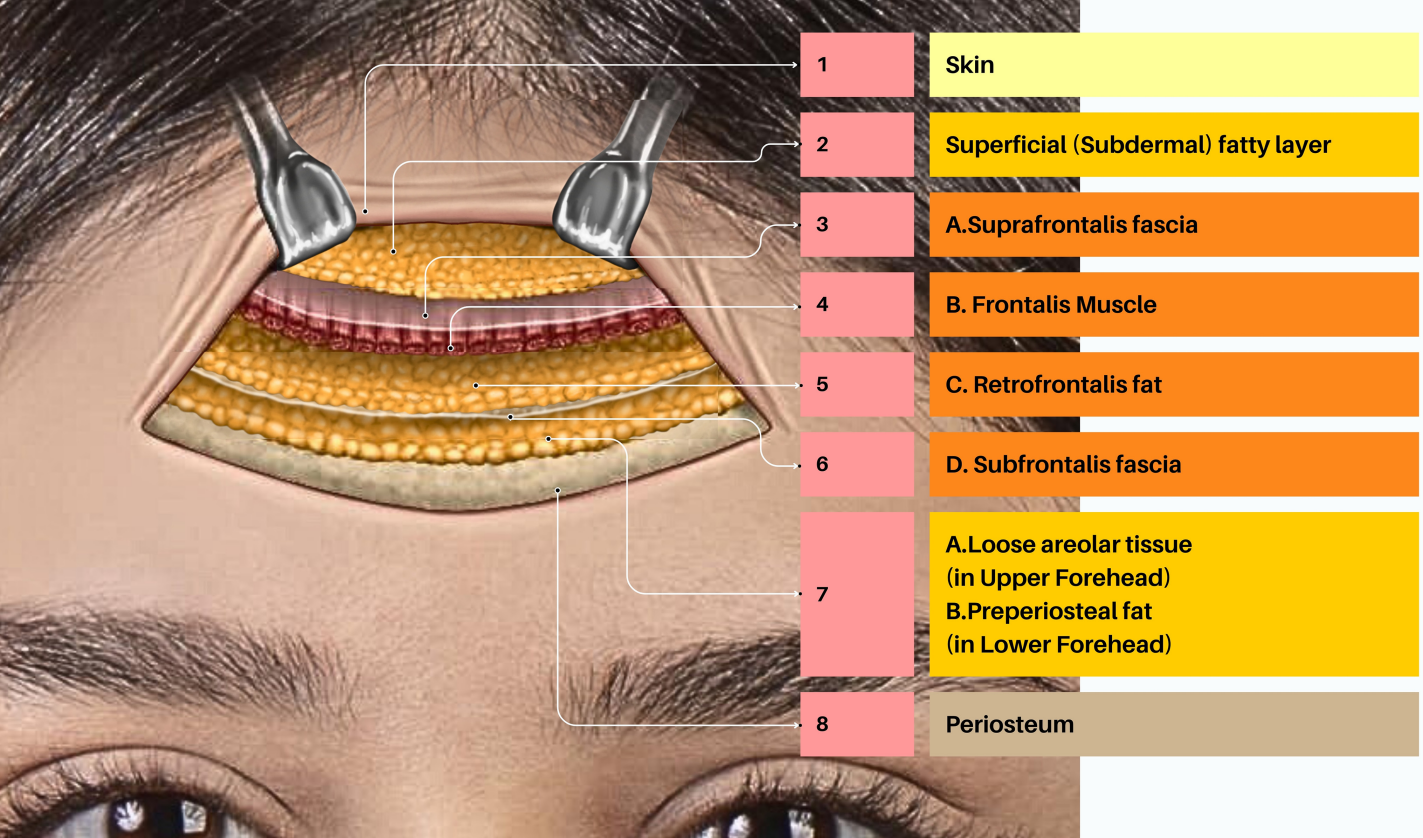
Anatomical layers of the forehead. The anatomical layers of the forehead: (1) skin; (2) superficial fat; (3) suprafrontalis fascia; (4) frontalis muscle; (5) a homogenous fat layer (separated by the orbicularis retaining ligament and supraorbital ligamentous adhesion into preseptal, retro‐orbicularis, and retrofrontalis fat); (6) subfrontalis fascia; (7) a compartmentalized supraperiosteal plane containing loose areolar connective tissue and preperiosteal fat within the prefrontal space; and (8) periosteum.^8^ Image courtesy of Dr. Ahmad Nazari.

Forehead anatomy also exhibits significant sexual dimorphism, and this must be considered when planning aesthetic treatment. For example, the female hairline tends to show a descending slope from the medial brow to the brow peak and a rounded shape, whereas males typically have a horizontal or ascending hairline slope resulting in a rectangular or “M” shape.^9^, ^10^ This difference should caution against over‐lateral forehead contouring in females, which can inadvertently yield a squared off (masculine) appearance that diverges from usual aesthetic preferences.^11^ In addition, the frontal region in females often has a pronounced eminence, whereas in males this area is more oblique and lacks the eminence.^12^

Key differences between racial groups should also be considered. For example, disparities in underlying skeletal support and in the propensity of soft tissue to sag mean that facial aging is typically slower in Asian people versus Caucasians.^13^

### 2.2 Assessments

From a profile perspective, the key assessment in the forehead region is concavity. Photonumeric tools are often used in clinical trials and can sometimes be useful in routine practice—particularly for less experienced injectors. Such an instrument has been developed for classifying forehead concavity on a scale from 0 (round and convex) to 4 (severely concave) (Figure 2A).^14^

**FIGURE 2:**
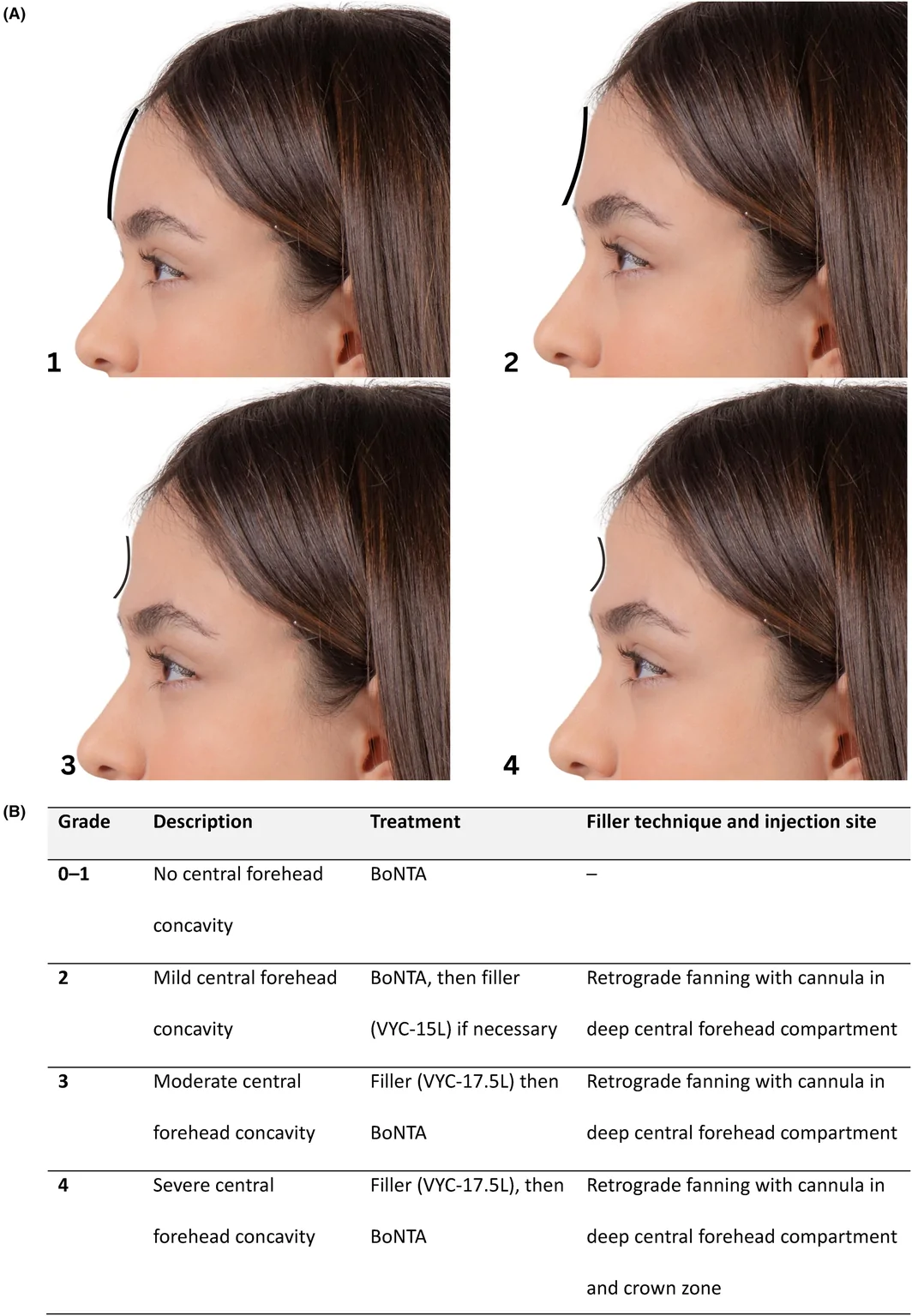
Forehead concavity grading and treatment approach. Concavity is graded on a five‐point scale (A) from 0 to 4: 0, round convex (devoid of concavities and projecting slightly forward when viewed in profile); (1) flat (extending from the frontal eminence to the supraorbital ridge without any intervening depressions); (2) mild concavity (presence of mild depression between the frontal eminence and the supraorbital ridge); (3) moderate concavity (presence of moderate depression in the said region); (4) severe concavity (presence of severe depression in the said region, representing frontal bossing in its severest form). Images courtesy of Dr. Lisandro Farollch‐Prats. The proposed treatment approach (B) is based on this grading system. BoNTA, botulinumtoxinA.

Three‐dimensional imaging systems can be used to assess pre‐treatment volume deficit and then to quantify post‐injection volume replacement. They also allow for sophisticated vectorial analysis of the tightening and elevation of the forehead, and can be used to quantify changes in key angles on the face. These evaluations may be relevant both to the forehead and to other facial areas discussed later in this paper.

### 2.3 Treatment techniques

A strategic approach to forehead rejuvenation based on concavity grading is provided in Figure 2B. This employs botulinumtoxinA (BoNTA) to smooth out frown lines, as well as fillers to volumize in the central area in patients with mild–severe concavity (Grades 2–4).

With onabotulinumtoxinA, the approved dose is 20 U administered across five injection sites.^15^, ^16^ This regimen was efficacious and safe in Phase 3 clinical trials when combined with treatment of glabellar lines (20 U).^17^, ^18^ To reduce the risk of eyebrow ptosis, injections should be focused on the upper part of the forehead, where the frontalis muscle acts primarily as a hairline depressor rather than an eyebrow elevator^19^; practitioners are also advised not to exceed the recommended dose.^20^ Results may be optimized by combining with injection of lateral canthal (crow's feet) lines at the approved dose (24 U).^18^

Treatment of the forehead with fillers is a more advanced procedure and should only be attempted by adequately trained practitioners. The key safety concern is to avoid injection into the supratrochlear and supraorbital arteries. Both are branches of the ophthalmic artery, and hence erroneous administration of filler can have catastrophic consequences, including blindness.^21^, ^22^

A recent ultrasound‐based study found that the deep branches of the supratrochlear and supraorbital arteries change anatomical plane from deep to superficial to the frontalis muscle just above the superior orbital rim.^23^ This suggests that filler can be administered into the supraperiosteal plane.^23^

The product used should have a medium–high elastic modulus (G′) and low–medium cohesivity to facilitate molding and a small amount of spread following injection.^24^ Appropriate soft and moldable hyaluronic acid (HA) fillers that can adapt to such a dynamic and delicate area include VYC‐15L and VYC‐17.5L.^25^

Injections should preferably be made using a large‐diameter, blunt‐tipped cannula (22–25 G), based on making contact with the bone and advancing the device within this plane to the treatment site. This helps to ensure precision and minimize risk—as demonstrated in a study showing better control of filler placement with cannulas compared with needles.^26^ The use of a retrograde fanning technique also facilitates precise and even filler deposition. For individuals with severe (Grade 4) concavity, treatment may extend to the crown zone, necessitating a high level of technical skill.

Treatment specifications can be divided according to two main patterns of forehead concavity: superior and inferior (Figure 3). For the superior type, the location of concavity is within the upper region of the deep lateral forehead compartment. Here, the preferred cannula entry point is 1 cm below the hairline and 0.5 cm medial to the temporal crest, with 0.3–0.5 mL of product administered into the supraperiosteum. For the inferior type, treatment requires precise targeting and administration into the prefrontal fat pad compartment, particularly focusing on the deep lateral section of the inferior forehead. The cannula entry point is 0.5 cm above and medial to the junction of the superior and lateral orbital rim. Around 0.3 mL of product should be administered into the supraperiosteum.

**FIGURE 3:**
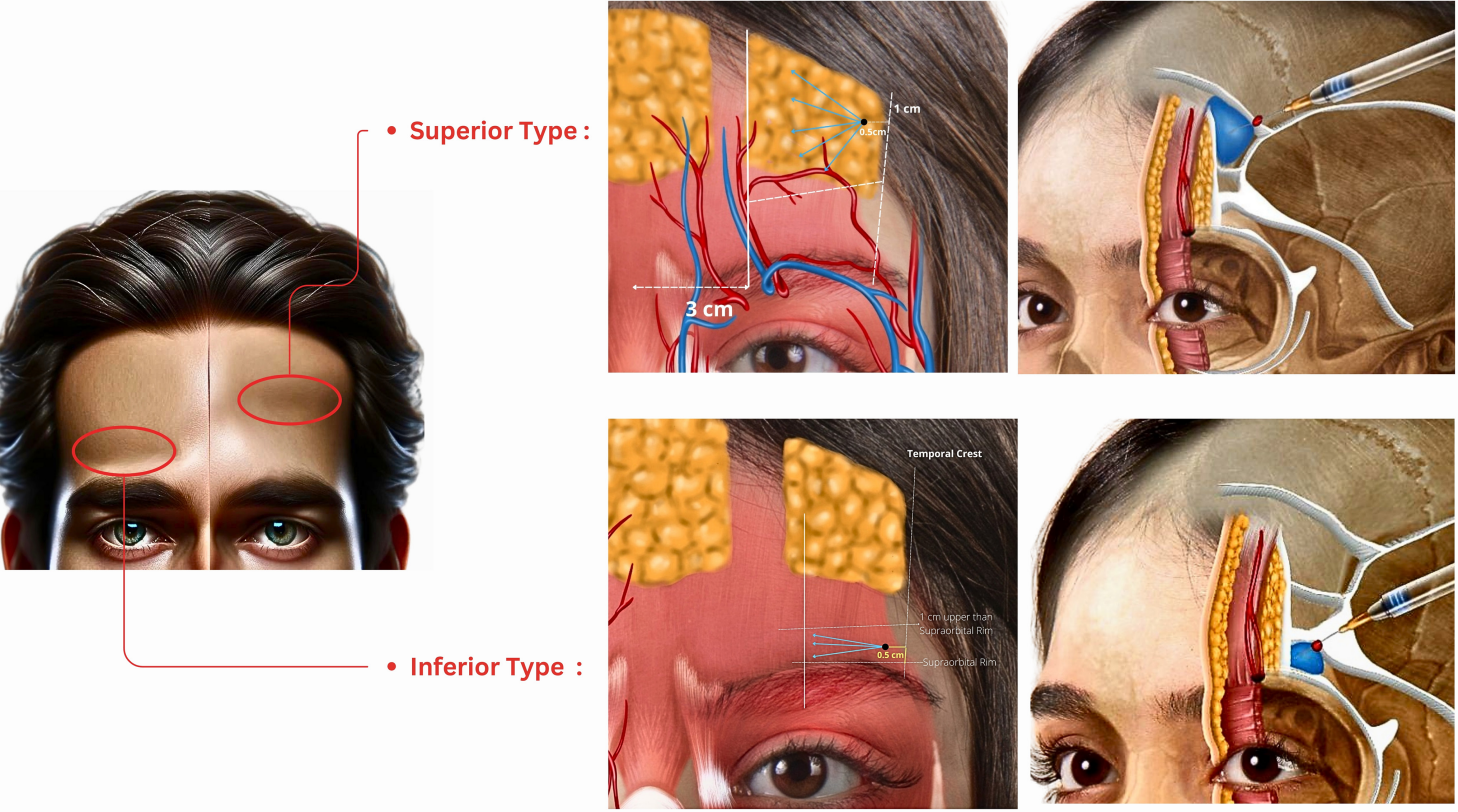
Correction of superior and inferior types of forehead concavity. For the superior type of forehead concavity, the lateral boundary is the temporal crest, the medial boundary is a line perpendicular to the supraorbital foramen or a point 3 cm lateral to the midline of the forehead, the superior boundary is the hairline, and the inferior boundary is the middle forehead septum, extending from 3 cm above the superior lateral rim of the orbit to 1.5 cm above the glabella. For the inferior type of forehead concavity, the lateral boundary is the temporal crest, the medial boundary is situated laterally to the supraorbital artery, the superior boundary is 1 cm above the orbital rim, and the inferior boundary is the superior rim of the orbit. In cases of superior concavity, the preferred cannula entry point is 1 cm below the hairline and 0.5 cm medial to the temporal crest; for inferior concavity, the cannula entry point is 0.5 cm above and medial to the junction of the superior and lateral orbital rim. Filler should be administered into the supraperiosteum using a retrograde fanning technique. Images courtesy of Dr. Ahmad Nazari.

## 3 NOSE

### 3.1 Anatomy

Filler use in the nose requires high levels of training, advanced technical skills, and extensive anatomical knowledge. As with the forehead, key safety considerations in this area include a comprehensive understanding of tissue layers and appreciation of the likely locations of key arteries.

With regard to the former, a recent ultrasound imaging study observed a five‐layer arrangement at the mid‐nasal dorsum, consisting of: (1) skin; (2) superficial subdermal fat; (3) the nasalis muscle; (4) loose areolar connective tissue, and (5) perichondrium.^27^ By contrast, the nasal radix and tip had no such layered arrangement of tissues. With respect to the arterial vasculature, the same study noted that this was superficial (subdermal) in most of the analyzed individuals: nasal radix, 91.7% of subjects; midnasal dorsum, 80.0%; and nasal tip, 98.3%.^27^

A key change to nasal anatomy that occurs as we age is a widening of the pyriform aperture.^28^ This may manifest outwardly as a broadening of the alar base, drooping of the nasal tip, and an apparent increase in the length of the nose.^29^

Injectors should also be wary of potential anatomical changes resulting from previous surgery to the nose.

### 3.2 Assessments

The Golden Ratio (also known as Phi)—with a value of 1.618—is a commonly occurring proportion in nature that seems to be aesthetically pleasing both in general and when applied specifically to facial features.^30^, ^31^ Thus, if the intercanthal distance is set to 1.0, other key parameters might be as follows:

- Nasal length (lash line to columella), 1.618.
- Alar base width, 1.0.
- Intercanthal line to nasal tip, 1.0.
- Dorsal width, 0.618.
- Tip height, 0.618.

Three‐dimensional imaging systems can be used to evaluate these distances before and after treatment.

From a practical perspective, other key nasal measurements that are relevant to profiloplasty are provided in Table 1. It is important to consider these aesthetic parameters according to the patient's gender. For example, the preferred vertical position of the nasion may be somewhat lower in females (at the level of the upper lid ciliary margin [CM] or the midpupil) compared with males (between the CM and supratarsal fold).^32^ A recent Chinese study also noted a preference for vertical positioning of the nasion at midpupil level in female faces.^37^ This difference between sexes has practical implications: when correcting a nasal hump using filler in women, it is particularly important to avoid going beyond eyelash height if possible.

**TABLE 1:** Key assessments of the nose.^32^, ^33^, ^34^, ^35^, ^36^

Measurement	Definition	Approximate aesthetic ideal
Nasofrontal angle	Angle between the glabella‐to‐nasion line and nasion‐to‐tip line	115°–135°
Nasofacial angle	Angle between the nasion‐to‐tip line and the plane of the face	~36°
Nasolabial angle	Angle between the columella line and philtrum line	95°–100° (males); 103–108° (females)
Nasion height	Anterior projection of nasion	10 mm anterior to the corneal plane
Nasion level	Vertical position of nasion	Between midpupil and CM (females); between CM and supratarsal fold (males)
Dorsum length	Distance from radix to tip	Equal to the distance between stomion and mentum
Tip projection	Proportion of nasal length	~60%

Abbreviation: CM, upper lid ciliary margin.

### 3.3 Treatment techniques

Novel fillers with greater cohesivity and increased G' have made nonsurgical rhinoplasty increasingly achievable. These products provide greater resistance to deformation and decreased lateral spread, allowing vertical projection to be maintained.^24^ For example, VYC‐20L has proven safe, effective and durable in both clinical trials^38^ and real‐world studies^39^, ^40^ for restoring and creating structure, and providing natural‐looking results.

Typical indications for profiloplastic treatment of the nose include dorsal hump, insufficient nasal tip projection, and nasolabial angle correction. However, owing to the presence of vascular structures—particularly in the nasal cartilage region—it is important to mitigate risk during these treatments.^41^ Inadvertent intra‐arterial administration can lead to rare but very serious complications, including blindness.^21^, ^22^

As already noted, the arterial vasculature is typically located in the subdermal plane,^27^ suggesting that deep injection may be safer. Thus, when treating the nasal dorsum with filler, we favor the use of a 25 G cannula inserted through the rhinion deep into the periosteum or perichondrium layer—which is typically relatively easy to locate. In this way, the dorsum can first be augmented upwards, followed by entry through the same point downwards to the nasal tip. The technique is not difficult from a practical perspective but does require training to ensure proper placement. Bending of the cannula is typically not needed, owing to the natural delineation of anatomical layers at the rhinion. Overall, this method gives the practitioner the best chance of controlling the injection plane and preventing damage to the superficial arterial structures of the nasal tip. By contrast, when injections are performed from the tip, it is more difficult to stay within the “safe” layer, and the device may intrude into other levels.^41^

Injectors should also be aware that the course of the nasal arterial vasculature is most frequently lateral,^42^, ^43^, ^44^ and hence, where possible, fillers should be administered into the midline of the nose. From a technical perspective, it is important that practitioners use both hands. While one hand delivers the injection, the thumb and index finger of the other should first lift the soft tissue to make a suitable space for correct administration of filler.

Finally, at the base of the nose, injection of the pyriform fossa can be used to bring the nostrils closer together. Alongside filler placement in the columella, this can also act as a supporting “tripod” to facilitate upward rotation of the nasal tip.

## 4 LIPS

### 4.1 Anatomy

The lips and mouth are fundamental to human interaction. Indeed, the mouth is typically the focus of attention during social contact, while the smile plays a central role in facial expression and is a major determinant of attractiveness.^45^ However, a number of physical changes occur with age that can have a profound impact on overall perception. The key process of lip aging may be considered as a redistribution from “thickness” to “length.”^46^ Dimensional analysis of the upper lip in older (≥65 years) versus younger adults (≤30 years) found significant age‐related increases in length (women, 19%; men, 18%), decreases in thickness (up to 41% in women and 33% in men), and reductions in volume (women, 21%; men, 17%).^47^

Key underlying changes occurring with age include a fragmentation of structural components, an increase in subcutaneous adipose tissue, involution and reduced forward curvature of the orbicularis oris muscle, and thinning of the epidermis and dermis.^46^

These processes manifest outwardly as an increase in prolabial length, reduced pout, inversion of the vermilion, and general ptosis of the lips.^46^ In addition, the oral commissures typically turn downwards and “Cupid's bow” disappears.^46^, ^48^, ^49^

### 4.2 Assessments

Aesthetic improvement of the lips is primarily a matter of volume augmentation, with fuller lips typically considered more attractive.^50^, ^51^ Various photonumeric fullness grading instruments have been developed. For example, the validated Lip Fullness Grading Scale developed by Carruthers and colleagues uses a five‐point gradation from 0 (very thin) to 4 (full) (Table 2).^52^

**TABLE 2:** Key assessments of the lips.

Measurement	Approximate aesthetic ideal
Lip Fullness Grading Scale^52^	Five‐point photonumeric scale: 0, very thin; 1, thin; 2, moderately thick; 3, thick; 4, full
Relative proportions of upper: lower lip	1:1 when assessed using maximal height of each lip^53^; 1:1.6 to 1:2 when assessed at the midline^50^, ^54^, ^55^
Anterior projection	In Caucasians^56^: Relative to the S2 line: 8.8 mm (UL) and 6.7 mm (LL) in females; 10.3 mm (UL), and 7.8 mm (LL) in males Relative to the B line: 3.5 mm (UL) and 2.2 mm (LL)

Abbreviations: LL, lower lip; UL, upper lip.

It is also important to consider the relative proportions of the lips. A global survey of female preference found that an upper‐to‐lower lip ratio of 1:1 (assessed using the maximal height of each lip) was generally favored, regardless of cultural background.^53^ Measured at the midline, a ratio of 1:1.6 to 1:2 may be optimal.^50^, ^54^, ^55^

In addition, anterior projection must be considered. Both lips should project from the face—the upper lip slightly more than the lower one. Various reference lines can be used to assess this property. However, the most consistent may be Burstone's B line (running from the subnasale to the soft tissue pogonion) and Sushner's S2 line (running from the soft‐tissue nasion to the soft‐tissue pogonion).^56^, ^57^, ^58^ Estimated “normal” levels of lip projection relative to these two lines are summarized in Table 2.^56^

Mandibular positioning can be a factor in this. Retrognathia is typically associated with an everted lower lip and labial incompetence, whereas prognathism is more usually linked with upper lip retraction and thin vermilion.^59^

Finally, underlying dental factors should also be assessed. In particular, retraction of the cervical point of the upper incisor correlates with resulting retraction of the upper lip.^60^

### 4.3 Treatment techniques

The key purpose of treatment with fillers is to enlarge and reshape the upper and/or lower lips, thereby improving their aesthetic relationship with the nose, teeth, and other facial features.^52^ Fillers with subtle lift capacity are required,^61^, ^62^ but the exact product specifications may differ somewhat depending on the treatment goals. For example, a smooth and moldable filler with low–medium G' should be favored for subtle volumization, reshaping and defining of delicate areas; however, a product with medium–high cohesivity may be needed for correcting structural deficiencies and asymmetries or for greater volumization, although it should also be sufficiently soft to adapt to such a highly dynamic area.^24^ VYC‐15L and VYC‐17.5L have proven safety, effectiveness, and durability in clinical trials.^63^, ^64^, ^65^, ^66^

Understanding the likely locations of the superior and inferior labial arteries within the upper and lower lips, respectively, is crucial to safe treatment. However, their courses can be highly variable, as observed in a cadaver study of anatomical head specimens.^67^ Although the labial arteries were most often located in the submucosal plane (63.7%–86.0% of assessments), they sometimes ran intramuscularly (particularly in the midline area of the lower lip), and occasionally subcutaneously or in a combination of planes. Moreover, during their entire course within the upper or lower lip, the labial arteries changed plane at least once in around a third of all cadavers.^67^

With regard to injection depth, filler should be administered into the submucosa (dry mucosa), as this is likely to be the safest plane. Either a cannula or needle may be used depending on individual injector preference. To improve projection, the key areas to treat are the medial tubercle of the upper lip and the lateral tubercles of the lower lip.^68^

## 5 JAWLINE

### 5.1 Anatomy

A contoured jawline is essential to a facial profile that looks young and attractive.^69^ The mandible area is also a key determinant of sexual dimorphism. Outwardly, males typically have a sharp jawline, pronounced jaw angle, and a squarer face in general, whereas female faces are defined by an oval shape and a more delicate appearance to the mandibular pillar.^69^ Key skeletal differences include greater antegonial notch area, ramus length, and coronoid height in males versus females.^70^

Aging can have profound effects on the jawline. In particular, soft tissue changes such as skin atrophy and gravitational descent of mid‐facial fat contribute to marionette line formation, the appearance of jowls, and general loss of jawline definition.^71^ These effects may be worsened by the downward pull of depressor muscles.^71^ There are also important underlying bony changes. A computed tomography analysis noted significant age‐related decreases in ramus height and mandibular body height/length in both males and females, as well as increases in mandibular angle (measured at the inferior border of the mandibular body and the posterior border of the ramus).^72^ Aesthetically, this manifests as reduced definition of the lower border of the face and a “softer” overall appearance.^72^

### 5.2 Assessments

The mandibular angle typically increases over time and can be an important marker of profile alterations. In young adults, this angle is typically around 112° in males and 120° in females (Table 3), but it may increase by 10° or more with age.^72^

**TABLE 3:** Key assessments of the jawline and chin.

Measurement	Approximate aesthetic ideal
Jawline	
Mandibular angle	112° in males; 120° in females^72^
Cephalic index	75–85 indicates mesocephaly (a balanced head shape)^73^
Jawline Sagging Assessment Scale^74^	Five‐point photonumeric scale: 1, no sagging; 2, mild sagging; 3, moderate sagging; 4, severe sagging; 5, very severe sagging
Chin	
Facial angle	165°–175°^75^, ^76^, ^77^
Projection relative to the lower lip	As far as the lower lip in males; 1–2 mm behind the lower lip in females^69^
Chin Projection Assessment Scale^74^	Five‐point photonumeric scale: 1, ideal projection; 2, mild chin retrusion; 3, moderate chin retrusion; 4, severe chin retrusion; 5, very severe chin retrusion

In addition, the cephalic index can be a useful assessment tool. This is a simple measurement of overall head proportions, calculated as width divided by length, multiplied by 100.^73^ A value of <75 indicates dolichocephaly (a longer, narrower head shape); 75–85 indicates mesocephaly (a balanced head shape); and >85 suggests brachycephaly (a shorter, wider shape). Although these categories are nonspecific, they can have important aesthetic implications. For example, in dolichocephalic individuals, the mandibular rim is typically oblique, with a mandibular angle greater than 120°. In treating these patients, vertical improvement may be needed, based on enhancing the mandibular rim towards the inferior.

Soft‐tissue changes should also be assessed. An international expert group recently published a novel, validated five‐point photonumeric scale for evaluating sagging of the jowl, graded between 1 (no sagging) and 5 (very severe sagging).^74^ This tool may be helpful in obtaining an objective and reproducible assessment of jowl formation.

### 5.3 Treatment techniques

Improving the definition of the jawline may require a combination of bulk reduction and border augmentation.^78^ In some cases, surgical approaches will be required for full correction, but injectables are increasingly in demand as a less invasive alternative.

Techniques based on onabotulinumtoxinA injection are currently under investigation in Phase 3 trials of patients with masseter muscle prominence (NCT04073303, NCT06137287, and NCT06068855). A previous Phase 2b study provided promising safety and efficacy data,^79^ and this approach offers great potential for contouring of the lower face. Moreover, fillers are now well‐established for treatment of the jawline—typically using products with high G' and high cohesivity, which maximize volumizing capacity and resist shear and compression deformation.^80^ For example, the robust HA filler, VYC‐25L has proven safety, effectiveness and durability in both clinical trials^80^, ^81^, ^82^ and real‐world practice^83^, ^84^ for sculpting, contouring and definition of the jawline, with natural‐looking results. A novel hybrid filler that combines the immediate lifting capacity of HA with the collagen stimulation effects of calcium hydroxyapatite (CaHA) has also shown promising safety and effectiveness in early studies.^69^, ^85^, ^86^

When using fillers, the key safety consideration is avoidance of the facial artery and vein, which cross the mandible anterior to the masseter and are located in the deep fat compartment.^69^ Hence, this plane should be avoided when injecting. To further minimize the risk of vascular complications, we advocate the use of a 22 G blunt‐tipped cannula. This device also has advantages with regard to treating a large area via a single entry point and reducing bruising.^69^, ^87^

In patients with visible sagging of the jowl area, this is a sign of ptosis and excess volume and should not be treated with filler. However, in many cases, the shape of the jawline can be re‐established through treatment of the depressed area *medial* to the jowl. To do this, we favor a method based on first marking out an approximately rectangular treatment area based on the medial limit, labiomandibular sulcus, or medial border of the jowl, with the mandibular border as the inferior limit and the labial commissure as the superior limit.^69^ The cannula should be inserted lateral to the prejowl, and the product administered by retro‐injection into both the submuscular and superficial subcutaneous planes (Figure 4).

**FIGURE 4:**
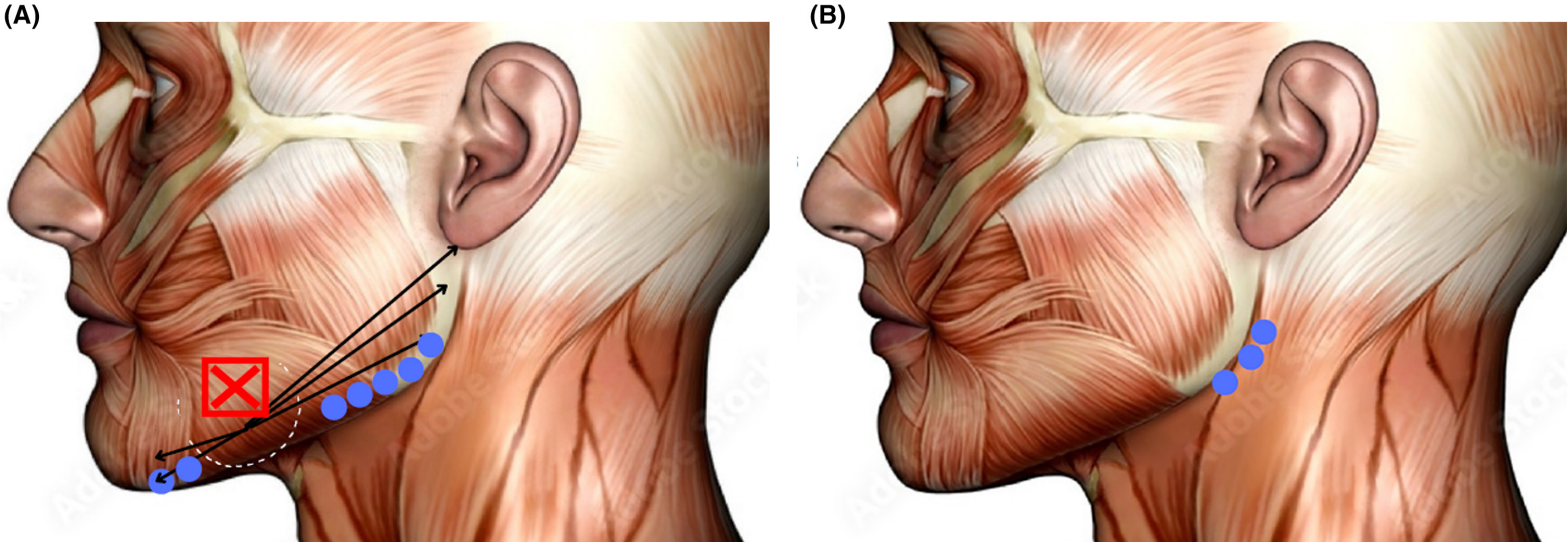
Filler treatment of the jawline (A and B). The cannula entry point is lateral to the prejowl area and can be used for filling the prejowl (forwards from the entry point), as well as the mandibular line, angle, and posterior ramus (backwards from the entry point). The product should be administered into the superficial subcutaneous plane using a retrograde technique. It is paramount not to inject into the jowl. After injection, the treated area can be shaped with moderate pressure using the fingers. Images courtesy of Dr. Lisandro Farollch‐Prats.

A second key area to treat is the mandibular line running posterior to the jowl up to the mandibular angle. The cannula can be inserted using the same entry point as for the prejowl area, with the product administered into the subcutaneous plane by retrograde injection.^69^ The filler should then be shaped using the fingers.

A third important zone is the mandibular angle and ramus. As with the prejowl, the injector should demarcate a rectangular treatment area—running vertically over the ramus based on two vertical lines ~3 cm in length and ~2 cm apart from each other.^69^ The cannula should be inserted at the mandibular angle, and the filler deposited subcutaneously by retrograde injection. The product can then be pressed onto the bone using the second finger to shape the final result.

Indirect effects on the lateral midface and lower face—including improvements in jawline contour—can also be achieved through temporal lifting.^88^ This procedure is based on filler injection into the subdermal superficial fatty layer. The preferred access point is the midportion of the zygomatic arch. The cannula should be advanced to a position oblique and posterior to the hairline, and once the total length of the device has been inserted, a product bolus can be administered, followed by subsequent massage.^88^

## 6 CHIN

### 6.1 Anatomy

The chin is a distinctive feature of the anterior mandible found only in *Homo sapiens* among all mammals.^89^ It is a key element of the human facial profile, and as with the jawline, is an important determinant of sexual dimorphism—male chins are typically significantly wider than those of females.^70^ Hypoplasia of the chin can have a significant impact on facial aesthetics in both men and women and is often seen as unattractive.^90^

Moreover, gradual loss of lateral and inferior volume means that the central chin area often appears to protrude more with age, while lateral atrophy causes ptosis that manifests as apparent widening of the chin.^91^

### 6.2 Assessments

Recession of bone in the mental area is a key cause of chin hypoplasia.^75^ This can be assessed using the “facial angle”—measured between a line connecting the glabella to the subnasale and a second line running from the subnasale to the pogonion (Table 3). It is easily calculated using three‐dimensional imaging technologies. An angle less than 165° indicates a retrognathic mandible, whereas an angle greater than 175° is suggestive of a deficient maxilla or prognathic mandible.^75^, ^76^, ^77^

When assessing the chin, it is also important to consider projection relative to the lower lip.^69^ Ideally, the male chin should project only as far as the lower lip, whereas in females, the lower lip should go 1–2 mm beyond the chin. To simplify the assessment process, an expert group recently developed a simple five‐point photonumeric scale for evaluating chin retrusion, rated from 1 (ideal projection) to 5 (very severe chin retrusion).^74^

### 6.3 Treatment techniques

As with treatment of the jawline, the use of fillers in the chin region typically requires products with high G' and high cohesivity. The robust filler VYC‐25L has been proven safe, effective and durable in clinical trials^80^, ^81^ and real‐world studies,^83^, ^84^ providing contouring and definition, with natural results. A novel HA–CaHA hybrid filler has also shown promising early results.^69^

Although this area is usually considered to be a relatively safe filling zone, particular care is needed to avoid penetrating the ascending mental artery, which crosses the chin towards the lower lip. A topographic anatomy study using a three‐dimensional camera found significant inter‐individual heterogeneity in arterial size but noted that there was typically a dominant ascending mental artery on one side of the chin (right, 61%; left, 39%) with a much smaller artery present on the other side.^92^ The dominant ascending mental artery was shown to enter the chin paracentrally—around 6 mm from the midline—within the muscular anatomical plane.

In general, the simple rule is that filler should mainly be placed anteriorly (i.e., in the pogonion) for augmenting chin projection, and inferiorly (i.e., in the menton) for increasing length.^69^ We favor the use of a 22–25 G blunt‐tipped cannula, based on a single entry point in the midline and multilayered product deposition. This should begin with administration into the supraperiosteal plane using a retrograde, lateral‐to‐medial tunneling technique, in order to improve the length, projection and rotation of the mentum.^93^, ^94^ More superficial subcutaneous injections can then be employed as needed to shape the final result.

When first treating patients in the chin area, it may be worthwhile to start with lower product volumes to avoid creating excessive pressure within tight tissue, and then to perform a second procedure if more is needed.

## 7 A HOLISTIC APPROACH TO PROFILOPLASTY

When undertaking profiloplasty with injectables, it is important to go beyond merely treating specific facial areas in isolation to consider the entire face. Assessment can be facilitated using key parameters introduced earlier in this paper, such as:

- Overall head proportions based on cephalic index (dolichocephaly, mesocephaly, and brachycephaly);
- Degree of maxillary retrusion; and
- The positional relationship of the jawline (particularly retrognathia and prognathism).

Although it is a relatively nonspecific instrument, cephalic index can have significant implications for overall treatment needs. Dolichocephaly (a long, narrow head shape) is more commonly seen in Caucasian patients and is associated with facial profiles that show a retrognathic chin, projected nose and concave forehead. In addition, the mandibular rim is usually oblique and the mandibular angle greater than 120°—such that vertical improvement is needed based on enhancement of the mandibular rim towards the inferior. By contrast, brachycephaly (a short, wide head shape) is more common in East Asian patients, and is associated with a broader, more square‐shaped face. Commonly observed features include a “duck‐like” appearance to the lips, insufficient nasal projection, deep nasolabial folds, flattened eyebrows, and lateral hooding—all of which can impact on the profile and may therefore require correction.

In patients with maxillary retrusion, it is often useful to treat the pyriform fossa with a filler. For correct placement, a horizontal line can be drawn level with the beginning of the nasal alar groove, with injections made at least 3 mm below this line. The filler should be administered directly onto the bone of the maxilla to provide support and enhance facial contours. This helps to reduce the nasolabial fold and provides lateral support for the lower nasal cartilage. In addition, it gives superior support to the upper lip, reducing concavity in that area. The upper lip can also be injected directly to enhance projection. However, treatment should not be made in the lower lip to maintain balanced projection between the two lips.

With regard to the jawline, an orthognathic profile is typically considered to be the most attractive, although a retrognathic profile may be more accepted in women than men whereas prognathism is more tolerable in males.^95^ Prognathism is typically associated with reduced facial convexity, resulting in a straight profile and apparent deficiency in the middle third of the face; by contrast, retrognathic profiles are linked with increased facial convexity as well as a short chin–neck distance (Figure 5).^95^ Chin retrusion and retrognathism can also result in greater effort of the depressor muscles to close the lips. This causes lower lip protrusion, a deep mental crease, and more pronounced marionette folds. The increased effort of the orbicularis oris muscle to close the lips often also leads to perioral lines on both the upper and lower lips. Correcting the position of the chin—as described in the previous section—can help to address these issues.

**FIGURE 5:**
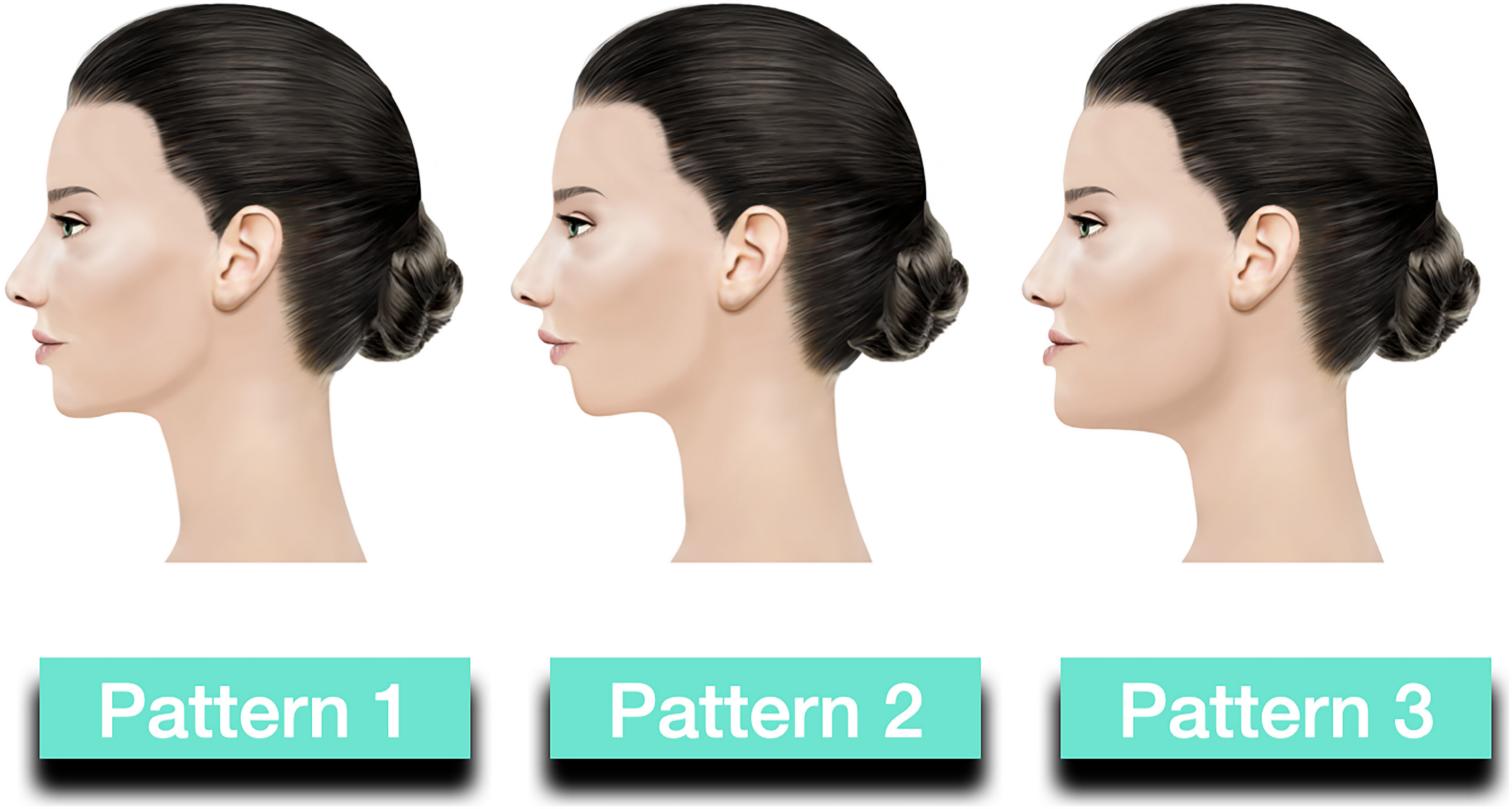
Positional relationship of the jawline. A normal or orthognathic jawline (pattern 1), a retracted or retrognathic jawline (pattern 2), and a protruding or prognathic jawline (pattern 3). Images courtesy of Dr. André Braz.

Figures 6 and 7 demonstrate the impact of full‐face profiloplasty in patient case studies.

**FIGURE 6:**
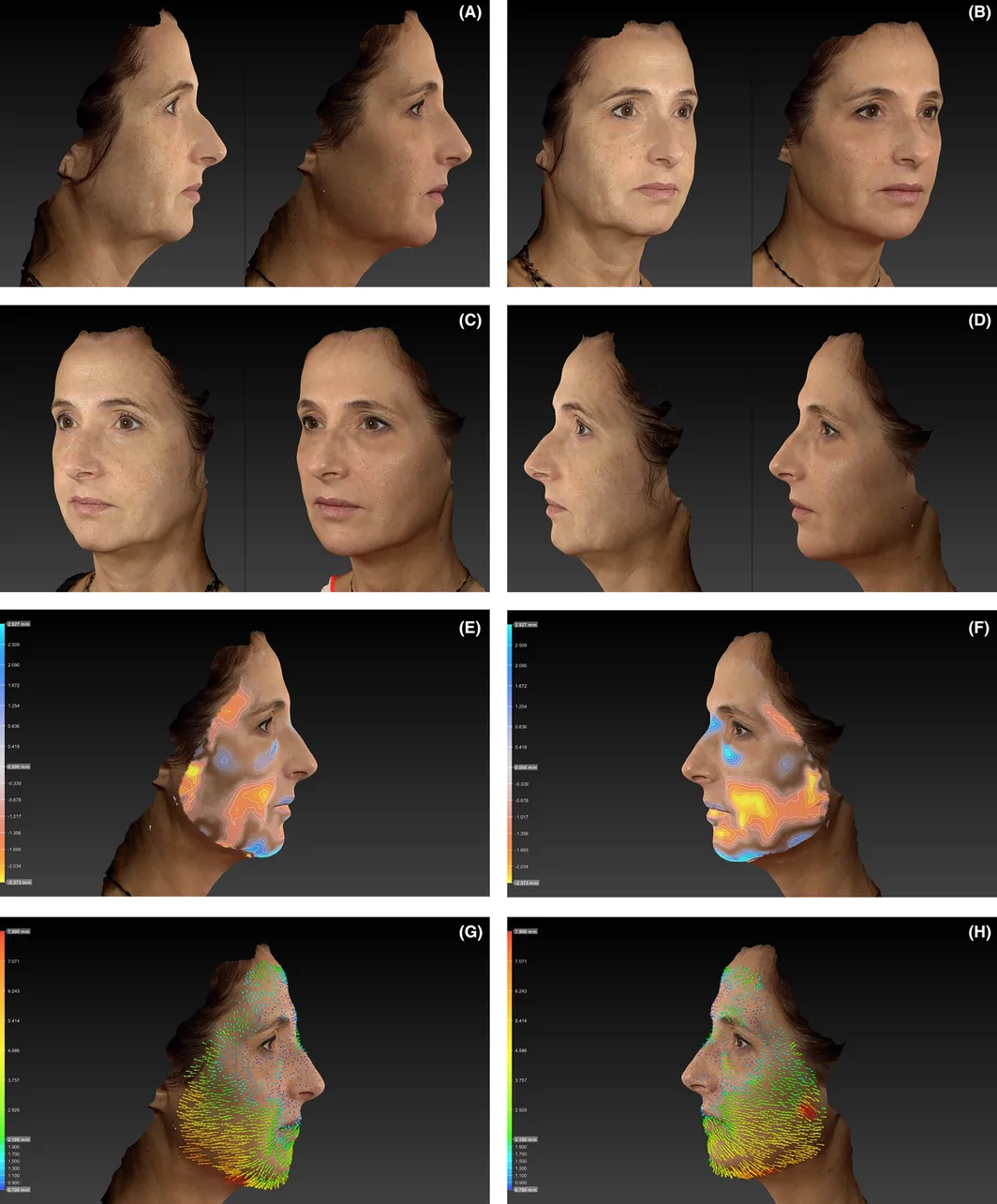
Profiloplasty with injectables. A 55‐year‐old female injected with 7 mL of hyaluronic acid filler: 0.65 mL of VYC‐20L in the left zygomatic temporal region and 0.6 mL in the right zygomatic temporal region; 1 mL of VYC‐20L in the left malar region and 0.75 mL in the right malar region; 0.4 mL of VYC‐17.5L in the upper lip and 0.1 mL in the lower lip; 0.5 mL of VYC‐17.5L distributed across the nasion and radix of the nose; 2.5 mL of VYC‐25L in the pre‐jowl and chin; and 0.25 mL of VYC‐25L in each mandibular angle. Parts A–D show the patient before (left) and 45 days after treatment (right). Analyses using a three‐dimensional imaging system highlighted post‐treatment changes in facial volume (parts E and F) and vectors (parts G and H). These assessments also demonstrated an increase in facial angle from 164° to 166° following treatment. Images courtesy of Dr. Lisandro Farollch‐Prats.

**FIGURE 7:**
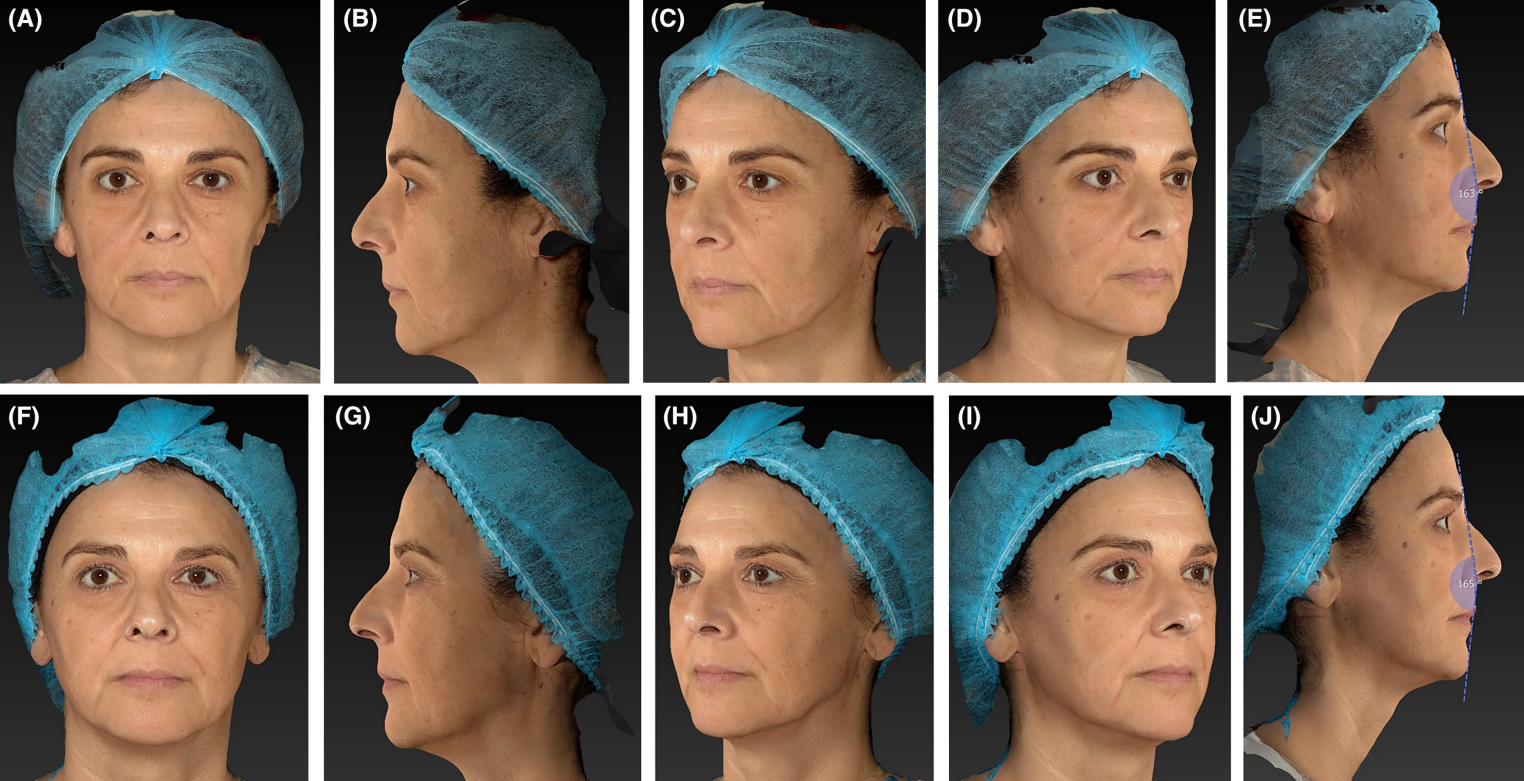
Profiloplasty with injectables. A 51‐year‐old female treated using HA fillers, a hybrid filler (combining HA with calcium hydroxyapatite), and botulinumtoxinA. She received 13.0 mL of HA fillers, equally distributed between the left and right sides: forehead, 0.8 mL of VYC‐17.5L; anterior temple, 1.4 mL of VYC‐25L; cheeks, 0.6 mL of VYC‐25L, 2.6 mL of VYC‐20L, and 1.6 mL of VYC‐17.5L; nasolabial folds, 2.0 mL of VYC‐17.5L; upper and lower lips, 1.0 mL (total) of Juvéderm Ultra 3; jawline, 2.0 mL of VYC‐25L; and chin, 1.0 mL of VYC‐25L. The patient was injected with 3.75 mL of hybrid filler in the preauricular area and the ascending ramus of the mandible (equally distributed between the left and right sides), as well as botulinumtoxinA for upper facial lines. Parts A–E show the patient before treatment and parts F–J show her at 1 year. Analyses using a three‐dimensional imaging system demonstrated an increase in facial angle from 163° to 165° following treatment (parts E and J). HA, hyaluronic acid. Images courtesy of Dr. Fernando Urdiales‐Gálvez.

## 8 CONCLUSIONS

Expansion of the injectable treatment armamentarium based on novel products has greatly improved practitioners' ability to reshape and enhance the facial profile. However, the concept of nonsurgical profiloplasty is still relatively new and remains insufficiently considered in routine practice. In this paper, we have provided a practical, user‐friendly approach to the assessment and treatment of key facial areas that constitute an attractive profile—and a more holistic view of how the profile can be improved for the benefit of our patients. This will be essential if we are to maximize the potential of full‐face treatment with injectables in the future.

## AUTHOR CONTRIBUTIONS

Lisandro Farollch‐Prats conceived the paper and was responsible for drafting of the initial version. All authors were involved in the interpretation of data for the work and in revising the manuscript critically for important intellectual content; gave final approval of the version to be published; and agreed to be accountable for all aspects of the work.

## FUNDING INFORMATION

Writing and editorial assistance was provided to the authors by Dr Timothy Ryder from Biological Communications Limited (London, United Kingdom) and funded by Allergan Aesthetics, an AbbVie company. Neither honoraria nor payments were made for authorship.

## CONFLICT OF INTEREST STATEMENT

Lisandro Farollch‐Prats, André Braz, and Fernando Urdiales‐Gálvez are consultants and speakers for AbbVie. Carola de la Guardia is an employee of Allergan Aesthetics, an AbbVie company. Ahmad Nazari reports no conflicts of interest to disclose.

## ETHICS STATEMENT

The authors confirm that the ethical policies of the journal, as noted on the journal's author guidelines page, have been adhered to. No ethical approval was required as this article includes no original research data.

## CONSENT STATEMENT

All of the patients whose photographs are used in this publication provided written informed consent.

## Data Availability

Data sharing is not applicable to this article as no datasets were generated or analyzed. AbbVie is committed to responsible data sharing regarding the clinical trials we sponsor. This includes access to anonymized, individual, and trial‐level data (analysis data sets), as well as other information (e.g., protocols, clinical study reports, or analysis plans), as long as the trials are not part of an ongoing or planned regulatory submission. This includes requests for clinical trial data for unlicensed products and indications. These clinical trial data can be requested by any qualified researchers who engage in rigorous, independent, scientific research, and will be provided following review and approval of a research proposal, Statistical Analysis Plan (SAP), and execution of a Data Sharing Agreement (DSA). Data requests can be submitted at any time after approval in the US and Europe and after acceptance of this manuscript for publication. The data will be accessible for 12 months, with possible extensions considered. For more information on the process or to submit a request, visit the following link: https://vivli.org/ourmember/abbvie/ then select “Home.”
